# Analysis of *al-2* Mutations in *Neurospora*

**DOI:** 10.1371/journal.pone.0021948

**Published:** 2011-07-19

**Authors:** Violeta Díaz-Sánchez, Alejandro F. Estrada, Danika Trautmann, M. Carmen Limón, Salim Al-Babili, Javier Avalos

**Affiliations:** 1 Department of Genetics, Faculty of Biology, University of Seville, Seville, Spain; 2 Faculty of Biology, Albert-Ludwigs University of Freiburg, Freiburg, Germany; University of Missouri-Kansas City, United States of America

## Abstract

The orange pigmentation of the fungus *Neurospora crassa* is due to the accumulation of the xanthophyll neurosporaxanthin and precursor carotenoids. Two key reactions in the synthesis of these pigments, the formation of phytoene from geranylgeranyl pyrophosphate and the introduction of β cycles in desaturated carotenoid products, are catalyzed by two domains of a bifunctional protein, encoded by the gene *al-2*. We have determined the sequence of nine *al-2* mutant alleles and analyzed the carotenoid content in the corresponding strains. One of the mutants is reddish and it is mutated in the cyclase domain of the protein, and the remaining eight mutants are albino and harbor different mutations on the phytoene synthase (PS) domain. Some of the mutations are expected to produce truncated polypeptides. A strain lacking most of the PS domain contained trace amounts of a carotenoid-like pigment, tentatively identified as the squalene desaturation product diapolycopene. In support, trace amounts of this compound were also found in a knock-out mutant for gene *al-2*, but not in that for gene *al-1*, coding for the carotene desaturase. The cyclase activity of the AL-2 enzyme from two albino mutants was investigated by heterologous expression in an appropriately engineered *E. coli* strain. One of the AL-2 enzymes, predictably with only 20% of the PS domain, showed full cyclase activity, suggesting functional independence of both domains. However, the second mutant showed no cyclase activity, indicating that some alterations in the phytoene synthase segment affect the cyclase domain. Expression experiments showed a diminished photoinduction of *al-2* transcripts in the *al-2* mutants compared to the wild type strain, suggesting a synergic effect between reduced expression and impaired enzymatic activities in the generation of their albino phenotypes.

## Introduction

Carotenoids are terpenoid pigments produced by photosynthetic organisms and many bacteria and fungi [1]. In plants and algae carotenoids serve as accessory pigments in photosynthesis [2] and in higher plants they are precursors of the phytohormones abscisic acid and strigolactones [3], [4]. Additionally, they provide attractive colors to many fruits and flowers. Animals get carotenoids through their diet and use them for the production of retinoids, among them the visual chromophore retinal [5] or the vertebrate morphogen retinoic acid [6]. Carotenoids are also responsible for the pigmentation of some birds, insects, fish, or crustaceans. Consumption of carotenoids has beneficial effects on human health [7], including protection against oxidative stress, cancer, sight degeneration syndromes and cardiovascular diseases [8].

**Figure 1:**
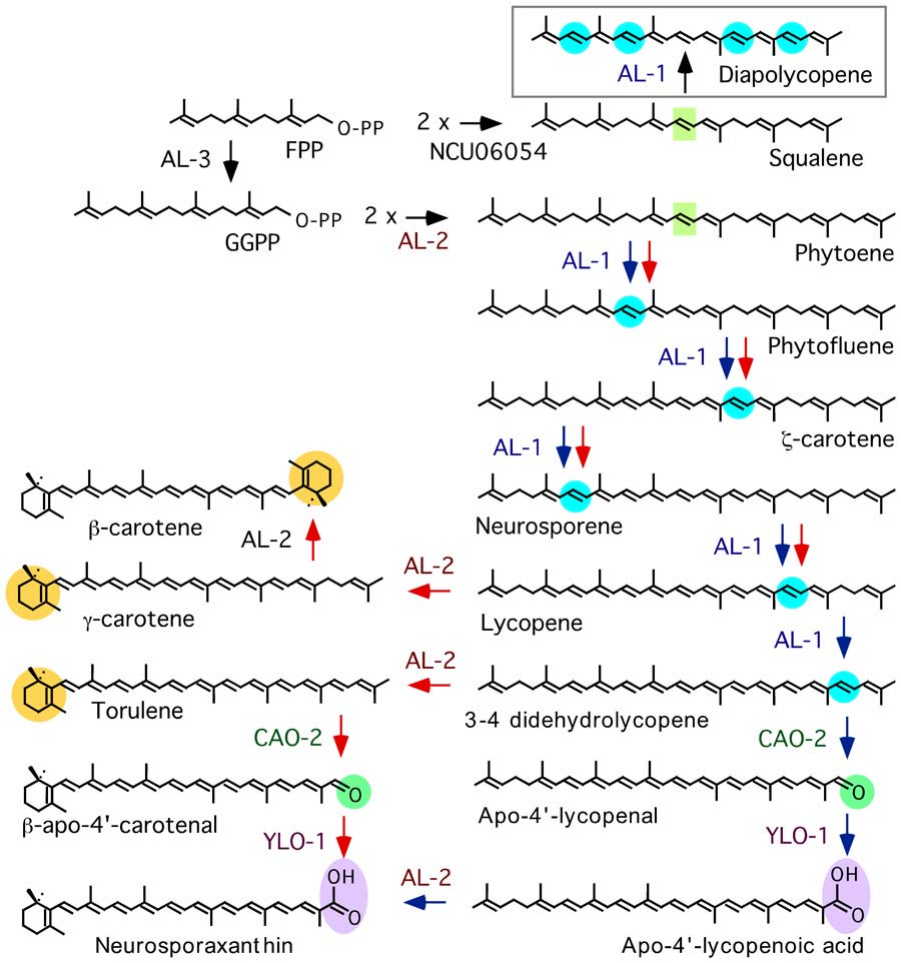
Carotenoid biosynthetic pathway of *N. crassa*. The gene products responsible for each enzymatic reaction are indicated. NCU06054 is the name annotated for squalene synthetase in the *N. crassa* genome. Chemical changes from precursor molecules are shaded with different colors. Blue and red arrows indicate the predominant steps from phytoene upon illumination at 8°C or at 30°C, respectively. Squalene synthesis is also depicted in the initial steps. The boxed reaction (diapolycopene synthesis) is proposed from the data.

Because of their laboratory amenability, microorganisms have been a major research system to investigate the biochemistry of carotenogenesis. Among them stand out several fungi, as the zygomycetes *Phycomyces blakesleeanus*, *Blakeslea trispora* and *Mucor circinelloides*, the basidiomycete *Xanthophyllomyces dendrorhous* or the ascomycetes *Fusarium fujikuroi* and *Neurospora crassa* [9], [10]. The carotenoid pathways of these fungi share the first biosynthetic steps, starting with the synthesis of geranylgeranyl pyrophosphate (GGPP) from farnesyl pyrophosphate (FPP) and the condensation of two GGPP units to produce the first molecule with a 40-carbon polyene chain, the colorless precursor phytoene (Fig. 1). The introduction of conjugated double bonds in the phytoene backbone yields molecules able to absorb visible light and provide the characteristic yellow, orange or red colors of the carotenoids. Subsequently, desaturated carotenes are targeted by one or two cyclization reactions, catalyzed by a cyclase enzymatic activity forming a β-ionone ring. Two cyclizations lead to β-carotene, the major end-product in zygomycetes. In *X. dendrorhous*, introduction of oxygen-containing functional groups in the β-ionone rings gives rise to astaxanthin. In *F. fujikuroi* and *N. crassa*, a carotenoid oxygenase cleaves the fully desaturated intermediate torulene, to produce a C_35_ apocarotenal [11], [12], which is further oxidized to produce the carboxylic xanthophyll neurosporaxanthin [13].

The knowledge on the fungal enzymes involved in these biochemical activities started with the identification of the genes responsible for the albino phenotype of the *N. crassa* mutants *al-1* [14], *al-2* [15] and *al-3* [16]. Thereafter it was shown that the encoded enzymes, AL-1, AL-2 and AL-3, respectively represent the dehydrogenase responsible for all the desaturation steps in the pathway [17], the phytoene synthase mediating the formation of phytoene from GGPP and the prenyl transferase catalyzing the synthesis of GGPP from farnesyl pyrophosphate [18]. For many years, the identification of the gene encoding the lycopene cyclase in *N. crassa* was elusive due to the absence of mutant phenotypes lacking this activity. However, the discovery of cyclase activity in the phytoene synthase CrtYB [19], the AL-2 counterpart in *X. dendrorhous*, pointed to AL-2 as a bifunctional protein with phytoene synthase (abbreviated hereafter PS) and cyclase activities. In CrtYB, the cyclase activity is located in the highly hydrophobic amino region of the protein, while the PS activity is located in the more hydrophilic carboxy region. The bifunctional nature of fungal PS genes was later demonstrated for the orthologous genes *carRP* and *carRA*, from the zygomycetes *M. circinelloides* [20] and *P. blakesleeanus* [21], and for *al-2* in *N. crassa* [22]. In contrast, cyclization of lycopene in plants and bacteria is exerted by independent enzymes encoded by different genes.

Cyclase deficient mutants of zygomycetes exhibit a red pigmentation due to the accumulation of lycopene, distinguishing them from β-carotene-accumulating, yellow wild type [20], [23], [24]. Genetic data in *P. blakesleeanus* suggested that PS and cyclase activities of CarRA are exerted by two independent polypeptides, CarR and CarA [23], and a conserved protease cleavage site was found in the boundary between both protein domains [21]. In *N. crassa*, only two reddish mutants affected in the AL-2 cyclase activity have been described so far [22], which accumulate the acyclic form of neurosporaxanthin [25]. One of them is probably affected also in the PS activity, as indicated by its lower carotenoid content [22]. This mutant phenotype suggested that both enzymatic activities are not physically separated in *N. crassa*. In this study, we determined the sequence of nine *al-2* mutants from the Fungal Genetics Stock Center [26] collection and analyzed their carotenoid content. Our data showed that one of the strains showing a reddish phenotype, similar to that of formerly described mutants, is affected in the cyclase domain, while the remaining eight strains are albino and are mutated in the *al-2* segment coding for the PS domain. Interestingly, the albino mutants with the lowest carotenoid content present traces of a carotenoid-like compound tentatively identified as the C_30_-pigment diapolycopene. The same compound was also observed in an *al-2* knock-out mutant, indicating that some of the *al-2* strains analyzed contain null mutant alleles. In addition, we investigated the impact of truncations of the PS domain on the cyclase activity in two *al-2* mutants. In support of the role of AL-2 as a single bifunctional polypeptide, we observed absence of cyclase activity in one of the PS-truncated mutants, despite the predicted presence of an intact cyclase domain.

## Results and Discussion

### Carotenoid biosynthesis in *al-2* mutants

**Figure 2:**
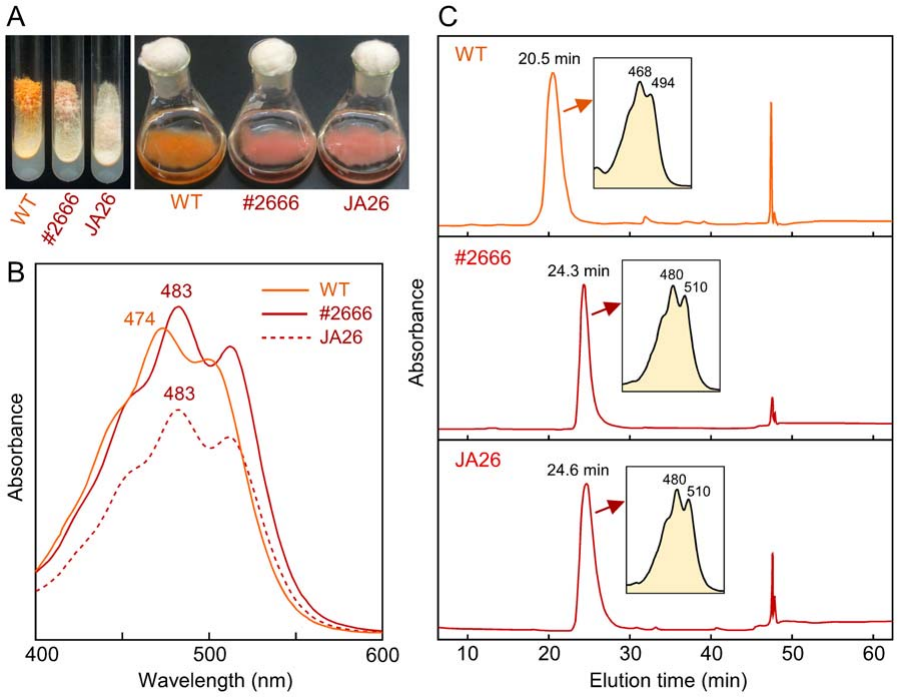
Phenotype of the mutant #2666. A: Left picture: slant cultures from the strain #2666 compared to the wild type and mutant JA26. The strains were grown for three days at 30°C in the dark and four days at 22°C under light. Right picture: flasks of the cultures used for the carotenoid analysis displayed in B and C. The cultures were incubated 48 h in the dark at 30°C and 24 h under illumination at 8°C. B: Absorption spectra of the crude carotenoid samples from the cultures of the wild type and the mutants JA26 and #2666. Peak wavelengths are indicated. C: HPLC profiles of the carotenoid mixtures from the samples shown on graph B. Absorption spectra of major peaks are shown in inner boxes.

Nine *al-2* mutants obtained from the FGSC collection were analyzed for carotenoid production. Eight of them exhibited an albino phenotype on slant cultures under light, while one of the strains, #2666, showed an apparent pigmentation, although less pronounced than that of the wild type (Fig. 2A). A detailed examination of the eight albino mutants revealed a very pale pigmentation in four of them, #900, #910, #913 and #4014, which was not noticeable in the other four mutants (Table 1). Neurosporaxanthin biosynthesis is particularly efficient in *N. crassa* upon illumination at low temperature, while illumination at the normal temperature results in a lower amount of this end product and a higher content of its carotene precursors (see, e.g., [13]). To learn more on the biosynthetic defects of the mutants, we investigated their ability to produce carotenoids under illumination at either 30°C or 8°C. As expected, the wild type strain accumulated more carotenoids at 8°C than at 30°C (Table 1), and the absorption spectrum was consistent with a higher proportion of neurosporaxanthin at the lower temperature. The mutant #2666 accumulated approximately 30% and 60% of the carotenoids produced by the wild type at 8°C and at 30°C, respectively (Table 1). However, the absorption spectrum (Fig. 2B) of the carotenoid from #2666 was different from that of neurosporaxanthin, accumulated in wild type, and resembled that of apo-4′-lycopenoic acid, the acyclic counterpart of neurosporaxanthin. A comparison with the spectrum of apo-4′-lycopenoic acid produced by the cyclase mutant JA26 [25] grown in parallel suggested that accumulation of this pigment in #2666, which is responsible for the similar reddish color of both strains. To further confirm the identity of the carotenoid produced by #2666, HPLC analysis of extracts from #2666, JA26 and wild type strains was performed. As shown in Fig. 2C, the pigments accumulated in #2666 and JA26 were identical, confirming the occurrence of the same biochemical defect in the carotenoid pathway in both mutants, i.e., loss of the cyclase activity [22], [25].

**Table 1:** Pigmentation and carotenoid content of the wild type and *al-2* mutants.

Strain^a^	Pigmentation^b^	Carotenoids^c^ (mg g^−1^ dry wt)	
		30°C	8°C^d^
Wild type	+++++	341±27	502±27 (Nx)
#896	−	∼0.5±∼0.2	∼1.4±∼0.1 (*)
#897	−	∼0.7±∼0.2	∼1.6±∼0.1 (*)
#900	+	11.4±0.2	5.6±0.6 (Nx)
#904	−	∼0.8±∼0.4	∼2.2±∼0.1 (**)
#910	+	10.5±0.2	∼2.4±∼0.8 (Nx)
#913	+	7.7±1.0	7.2±0.8 (Nx)
#914	+/−	4.0±0.1	5.6±0.8 (Nx)
#2666	+++	216±27	156±15 (La)
#4014	+/−	5.7±0.6	11.4±0.6 (Nx/La)

a Wild type: Oak Ridge 74-OR23-1A.

#indicates FGSC numbers for *al-2* mutants.

**Figure 3:**
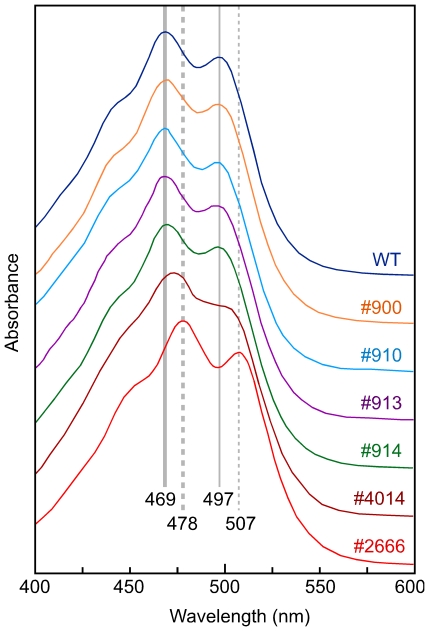
Spectra of the major carotenoid produced by the wild type strain, the reddish mutant #2666 and five leaky *al-2* mutants. The cultures were incubated 48 h in the dark at 30°C and 24 h under illumination at 8°C. Their carotenoids extracts were concentrated and separated by HPLC and spectra of the major peaks were displayed. Maximal and secondary absorbance peaks for neurosporaxanthin (469 and 497 nm) and apo-4′-lycopenoic acid (478 and 507 nm) are indicated by continuous and dashed grey bars, respectively.

b Appearance on agar slants under light at room temperature.

c Average and standard deviation from at least two determinations.

d Major carotenoid in parentheses (see Fig. 3); (Nx): neurosporaxanthin; (La): apo-4′-lycopenoic acid; (Nx/La) spectrum data consistent with a mixture of neurosporaxanthin and apo-4′-lycopenoic acid; (*): spectrum data consistent with diapolycopene; (**): not determined.

The amounts of carotenoids in the albino mutants were consistent with their pigmentation patterns (Table 1). The pale color observed in #900, #910, #913 and #4014 cultures could be explained by the accumulation of minor amounts of carotenoids, ranging from 5 to 13 mg/g in different strains and illumination conditions. So, these strains would contain mutations in the *al-2* gene resulting in enzymes with a leaky activity. To find out if these AL-2 altered enzymes are affected in the cyclase activity, the trace carotenoids accumulated at 8°C were separated by HPLC and the peak spectra were analyzed (Fig. 3). The mutants #900, #910, #913 and #914 accumulated neurosporaxanthin, as compared to the wild type major product. The spectrum of the peak in mutant #4014 was consistent with a mixture of neurosporaxanthin and apo-4′-lycopenoic acid, suggesting the occurrence of a leaky cyclase activity.

**Figure 4:**
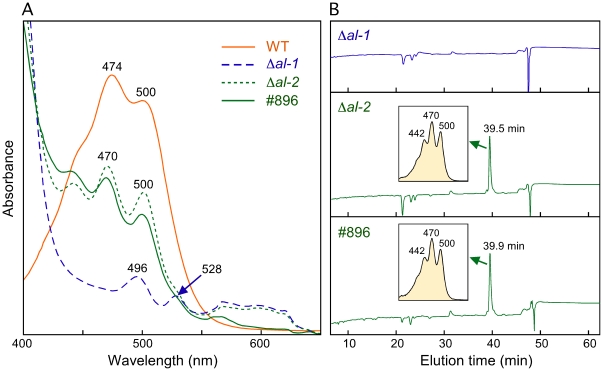
Detection of a carotenoid-like pigment in *al-2* mutants. A: Absorption spectra of the crude carotenoid samples from the cultures of the mutants #896 and the null mutants Δ*al-1* and Δ*al-2* incubated 48 h in the dark at 30°C and 24 h under illumination at 8°C. Relevant peak wavelengths are indicated. The spectrum of the wild type sample (WT) diluted about 150 times is shown for comparison. B: HPLC profiles of the carotenoid mixtures from the samples of the mutants displayed on the left. Absorption spectra of the major peaks are shown in inner boxes.

Unexpectedly, the three albino mutants with no detectable color in the slant cultures (#896, #897 and #904, Table 1) contained trace amounts of a carotenoid-like pigment, synthesized at 30°C and 8°C. These results fit a previous study, in which minor amounts of some intermediate carotenoids were found in *al-2* mutants [27], indicating that the PS activity can be partially replaced by a different enzyme. To check this hypothesis, we analyzed the content of colored carotenoids in null mutants of the *al-1* and *al-2* genes, obtained through targeted replacement by a hygromycin resistance cassette (FGSC collection). One of the *al-2* mutants with the lowest carotenoid content and a highly defective PS domain (see next section), #896, was included in the analysis. Crude extract spectra from mycelial sample of the Δ*al-2* mutant showed the presence of trace amounts of a carotenoid-like pigment with a central peak of 470 nm and a secondary peak at 500 nm (Fig. 4A). The same pigment was found in #896, but not in the Δ*al-1* mutant. In the latter case, there were trace amounts of other compounds, whose absorption peaks suggest that they are unrelated to carotenoids. HPLC analyses of these samples confirmed the presence of the carotenoid-like pigment in Δ*al-2* and #896 and its absence in Δ*al-1* (Fig. 4B).

### Carotenoid biosynthesis without phytoene synthase

The synthesis of detectable amounts of a carotenoid-like pigment in the absence of PS function (Δ*al-2* and #896 mutants) could be due to a residual PS activity by squalene synthase: the condensation of two FPP molecules catalyzed by this enzyme is highly similar to the condensation of two GGPP molecules achieved by phytoene synthase, differing only in the length of substrate and product (Fig. 1). Supportingly, the squalene synthase inhibitor squalestatin is also active on phytoene synthase [28], indicating that both enzymes have similar catalytic sites. Phytoene synthesis by squalene synthase was formerly proposed to explain the accumulation of minor amounts of lycopene in a *P. blakesleeanus* mutant with a premature stop codon in the cyclase domain of gene *carRA* [21], which was predicted to lack the PS domain. In our case, because of the lack of cyclase activity of the Δ*al-2* mutant, we would expect the synthesis of minor amounts of apo-4′-lycopenoic acid, which is the same carotenoid produced by the cyclase mutants #2666 and JA26. This hypothesis is contradicted by the chromatographic and spectrophotometric properties of the compound identified in the HPLC analysis, which do not correspond to those of apo-4′-lycopenoic acid (compare Fig. 2C and Fig. 4B). Moreover, the shape of the absorption spectrum and the maximal absorption peaks are highly coincident with those of diapolycopene [29], [30]. This C30 apocarotenoid, naturally produced by some bacteria, results from the introduction of four desaturations in the squalene molecule (diapophytoene) by the desaturase CrtN [31].

In conclusion, our data suggest that a minor proportion of squalene is desaturated by *al-1* to produce diapolycopene (Fig. 1). This compound would not be recognized as a substrate by the AL-2 cyclase domain, as indicated by the accumulation of the same compound in the mutant #896, which conserves this enzymatic activity. The extremely low amounts of this compound, insufficient to provide an externally detectable coloration, are probably explained by the lack of squalene accessibility to Al-1 in the cell. In support to this hypothesis, the syntheses of sterols and carotenoids were formerly found to occur in separate cell compartments in the fungi *P. blakesleeanus* [32] and *F. fujikuroi* [33]. Biochemical assays with purified squalene synthase and phytoene desaturase enzymes will be needed for future evaluation of their respective capacities to convert GGPP into phytoene in one case, and squalene into diapolycopene in the other.

### Mutants with predicted AL-2 truncated polypeptides

**Figure 5:**
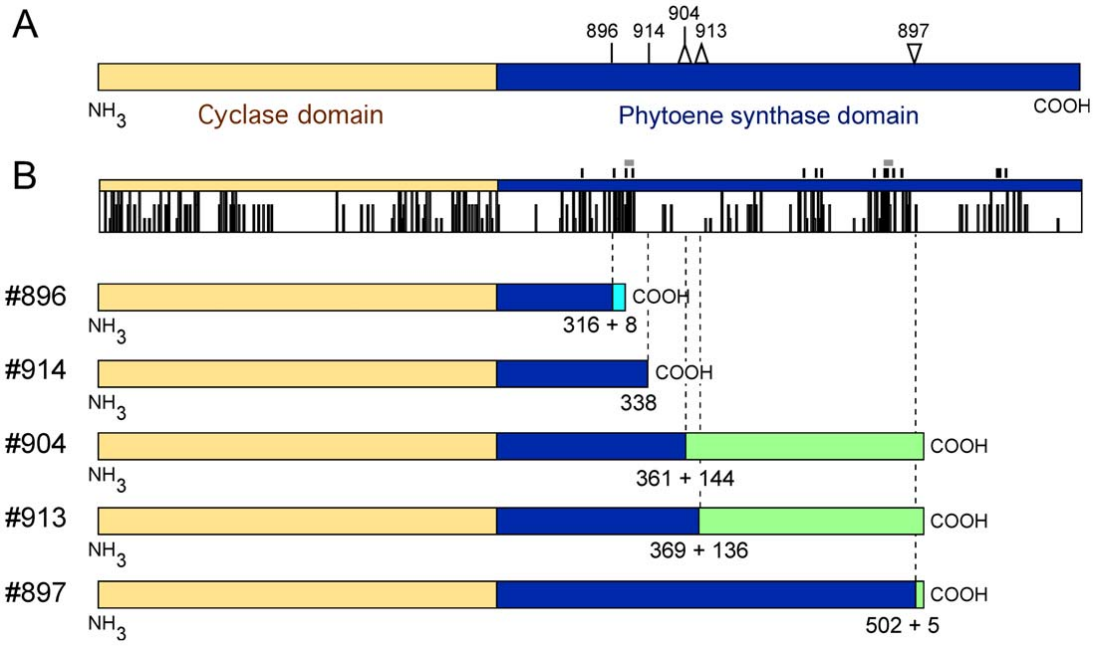
Predicted AL-2 truncated polypeptides in the mutants #896, #897, #904, #913 and #914. A: Scheme of the AL-2 wild type polypeptide and locations of the different mutations indicated in the text. The cyclase and phytoene synthase domains are indicated in orange and blue colors, respectively. Vertical bars: substitutions; upside triangles: small deletions; downside triangle: small insertion. FGSC numbers of the mutants are indicated for each mutation. B: Above: Map of conserved residues upon Clustal comparison of AL-2 from *N. crassa* (accession number L27652), with the cyclase/PS enzymes CarRA from *F. fujikuroi* (AJ426417) and *P. blakesleeanus* (CAB93661), CarRP from *M. circinelloides* (AJ250827), CrtYB from *X. dendrorhous* (AAO47570), and Car2 from *U. maydis* (UM06287). Full bars in the scheme indicate identical positions in the six proteins, 2/3 bars indicate conserved substitutions, and 1/3 bars indicate semi-conserved-substitutions (“*”, “:” and “.” symbols in the Clustal alignment, respectively). Small vertical bars over the scheme indicate amino acids involved in phytoene synthase catalytic domain. The small grey boxes indicate two short amino acid segments associated with substrate-Mg2^+^ binding. Below, schematic representation of predicted AL-2 truncated proteins. Random amino acid segments of the same reading frame are indicated with the same color at the carboxy ends of the polypeptides. The numbers below indicate predicted protein lengths plus number of random amino acids in the cases of frame-shift mutations.

To understand the biochemical basis of the mutant phenotypes, the sequence of the *al-2* alleles from all the strains under investigation was determined (Supporting Information S1). Five strains, #896, #897, #904, #913 and #914, contained mutations expected to produce truncated AL-2 polypeptides (Fig. 5). The cyclase domain was formerly identified in the first 244 amino acids of the AL-2 protein [22], while the remaining 358 amino acids corresponded to the PS domain. The five predicted proteins contain an intact cyclase sequence and different segments of the PS domain.

In the mutant #896 the 5′ GT splicing sequence of the second intron was replaced by two adenines. Sequencing of the cDNA version of the #896 *al-2* mRNA showed that the intron was removed through an alternative GT sequence, located 4 bp downstream from the original 5′ GT sequence. This alternative splicing results in a frame-shift mutation, with an early stop codon in the new reading frame. The predicted AL-2 polypeptide in #896 only has 72 out of 358 residues of the PS domain, followed by eight random residues. Failure of intron splicing would result in a similar protein because of the occurrence of a stop codon in frame with the intron sequence. The mutant #914 is expected to produce a similarly truncated AL-2 protein, with only 94 amino acids of the PS domain. In this case, the reason is a G to A transition in the third position of a tryptophan codon, resulting in a premature stop codon.

The other three mutants contain frame-shift mutations produced either by short insertions or deletions (Fig. 5). In the mutant #904, a GAA sequence was replaced by AG, resulting in a predicted AL-2 protein with 117 residues of the PS domain, followed by 144 random amino acids. A similar truncated protein is expected for mutant #913, with a T deletion producing a 125 aa PS segment followed by 136 random residues, coincident with those of the #904 protein. Finally, the mutant #897 holds a CAAGA insertion producing an AL-2 protein with 72% of the total PS segment, plus five random residues. The stop codon used for this predicted protein is the same used in that of #904.

The amino acids involved in the catalytic PS activity (data from the conserved domain database, see material and methods) are spread along the PS segment, from residues 297 to 556 (Fig. 5B), and most of them coincide with those participating in the substrate binding pocket (data not shown). This segment also includes two short amino acid segments (small grey boxes in Fig. 5B) associated with substrate-Mg^++^ binding. Therefore, four out of the five AL-2 truncated proteins have lost most of the residues involved in substrate binding and catalysis, while one of them (#897) conserves a large portion of the PS domain. Unexpectedly, two mutants holding severe AL-2 truncations (#913 and #914) exhibit higher carotenoid contents than #897 (Table 1). Although the amount of carotenoids in these mutants is drastically reduced in comparison to the wild type, the modest amounts of carotenoids detected in #913 and #914 should be attributed to a leaky activity maintained by the truncated enzymes.

### Mutants with complete AL-2 polypeptides

**Figure 6:**
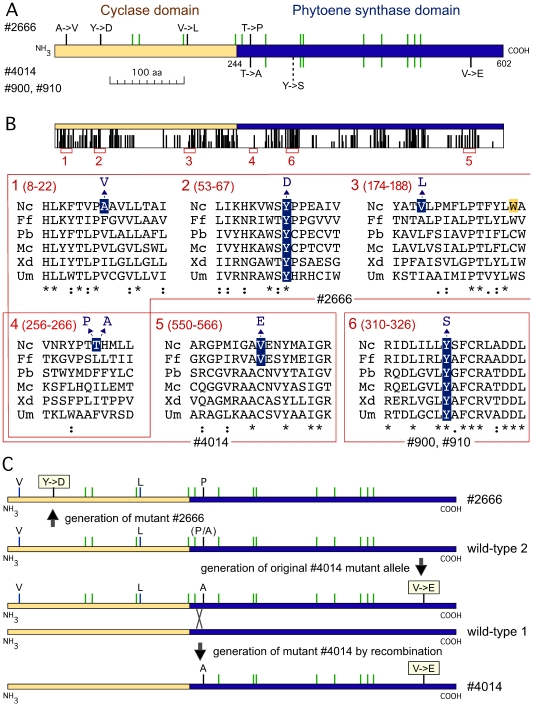
Point mutations in the *al-2* alleles in the mutants #2666, #4014, #900 and #910. A: Schematic representation of the mutations on the AL-2 polypeptide. Amino acid changes are indicated by black bars and silent mutations are indicated by green bars. Upper bars: mutations in strain #2666. Lower bars, mutations in strain, #4014. Dashed bar: mutation in strains #900 and #910. B: Comparison of the six polypeptide segments containing amino acid changes, taken from the Clustal analysis of AL-2 with the five fungal cyclase/PS enzymes mentioned in the legend of Figure 4. The location of the six protein segments are shown below the map of conserved residues. Numbers in parentheses indicate amino acids in the AL-2 protein. The sequences representing the mutations from each strain are boxed, and the affected residues are highlighted on blue background. In sequence number 3, the tryptophan affected in the mutant JA26 is indicated on yellow background. C: Proposed molecular events leading to the formation of the mutants #2666 and #4014. The X represents a cross-over in one of the backcrosses. Mutations are indicated with bars, as displayed in panel A. Relevant amino acid changes are boxed. Direct evidence for wild-type sequence is only available for wild type 1.

The remaining four mutants contained point mutations in their respective *al-2* alleles (Fig. 6). Two of them, #900 and #910, contained identical *al-2* sequences, with a single A to C transversion, which replaced a tyrosine residue by a serine (Fig. 6A). The affected tyrosine residue is involved in the catalytic site and it is highly conserved in fungal PSs, as shown by the comparison of examples from distant taxonomic groups: ascomycetes, basidiomycetes and zygomycetes (Fig. 6B). The occurrence of the same mutation in the two mutants could be coincidental or the result of a former misclassification.

The *al-2* alleles from the mutants #4014 and #2666 contained numerous point mutations compared to the standard wild type of *N. crassa*. Most of the mutations were silent, affecting third codon positions, and two mutations were located in the second intron, with no predictable consequences on intron splicing. Some of the mutations coincided in both strains, indicating a common origin (discussed below). Concerning the protein sequence, the predicted #2666 AL-2 polypeptide differed in 4 amino acids from the standard wild-type AL-2 sequence (Fig. 6A). Three changes (leading to V, L and P, Fig. 6A) affect non-conserved residues (mutations 1, 3 and 4 in Fig. 6B), and are unlikely to have significant consequences on AL-2 activity. In contrast, the fourth change replaced a highly conserved tyrosine residue by aspartic acid (Fig. 6A and mutation 2 in Fig. 6B). This mutation, located in the cyclase domain, is very likely to cause the carotenoid pattern exhibited by the mutant #2666, similar to that of JA26. The single mutation found in the JA26 *al-2* allele [22] affected a tryptophan residue close to the replacement of valine by leucine (mutation 3 in Fig. 6B, tryptophan indicated in grey); however, the latter mutation is unlikely to have an effect, as suggested by the occurrence of leucine at the same position in other fungal enzymes.

The AL-2 protein predicted for the mutant #4014 differs from the wild type enzyme in two amino acids, both of them located in the PS domain. One of the mutations affects the same base and amino acid than one of the mutations found in the strain #2666 (segment 4, Fig. 6A and B). The second mutation, more likely to produce the biosynthetic defect exhibited by the mutant, replaces a valine residue by one of glutamic acid. A valine was also found at this position in the *F. fujikuroi* enzyme, but the residue was a cysteine in the other enzymes checked. This residue is adjacent to a highly conserved alanine residue, involved in the catalytic site. The proximity of this mutation to the critical residue may explain the leaky phenotype exhibited by the mutant (Table 1).

The sequences of the *al-2* alleles from the mutants #2666 and #4014 suggest their origin from a different genetic background than that of the other mutants under investigation. The eight silent changes present in both mutant alleles compared to the standard wild type gene should be already present in the preceding wild type *al-2* allele. The original #4014 mutant was backcrossed seven times with the St. Lawrence wild type strain (data provided by the FGSC collection). It seems very plausible that the five additional silent mutations in the upstream *al-2* sequence identified in the mutant #2666 were also present in the original wild type strain. The current sequences are explained by the occurrence of an internal recombination in one of the backcrosses that led to the loss of these mutations (Fig. 6C). Probably, the valine and the leucine found in the cyclase domain of #2666 were not due to *de novo* mutations, but were present in the original wild type allele (wild type 2 in Fig. 6C). However, we cannot discard the possibility that the mutations were introduced by the UV mutagenesis process. Additionally, the available information indicates that either an A->P mutation occurred in the generation of #2666 or a P->A mutation occurred in the generation of #4014. We cannot distinguish between these two alternatives, but it seems evident that either proline or alanine were present in this wild type allele instead of threonine (mutation 4 in Fig. 6B).

### Cyclase activity of two AL-2 mutant enzymes

The carotenoid pattern of the mutant #2666 reveals a defect in the formation of cycled carotenoids, explained by the mutation in the cyclase domain of the *al-2* gene (Fig. 6). Additionally, this mutant produced less carotenoids than the wild type, pointing to a partial defect in PS activity. This effect could be related to the proline found at position 262, or it could be an indirect effect of the Y->D mutation in the cyclase domain. A lower carotenoid content is also exhibited by the mutant JA26, whose mutation was mentioned above. However, another mutant with a single mutation in the cyclase domain, JA28, exhibited defective cyclase activity, but accumulated similar amounts of carotenoids as the wild type [22]. These results suggest that certain mutations in the cyclase domain, but not others, interfere with the PS activity.

**Figure 7:**
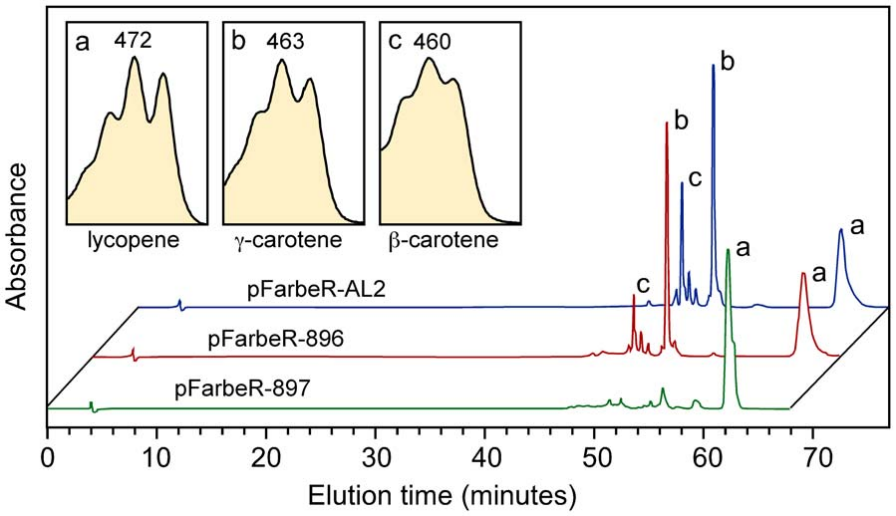
*In vivo* activity of AL-2 alleles in lycopene-producing *E. coli* strains. HPLC analyses of the carotenoids accumulated in *E. coli* cell expressing the genes *crtE*, *crtB* and *crtI* from *E. herbicola* and the *al-2* alleles either from the wild type (pFarbeR-AL2) or mutants #896 and #897 (pFarbeR-896 and -897, respectively). Absorption spectra of the major peaks and carotene identifications are shown in inner boxes.

To learn more on the crossed effects between the two AL-2 enzymatic functions, we investigated the cyclase activity of two mutants with truncated PS domains, #896 and #897, in comparison to the wild type. For this purpose, cDNA versions of the respective *al-2* alleles were cloned under control of the *lac* promoter in a plasmid with bacterial activities leading to lycopene production. Transformation of appropriate *E. coli* strains with the corresponding plasmids showed that the wild type AL-2 enzyme efficiently converted lycopene into γ-carotene and β-carotene (Fig. 7), demonstrating the functionality of the AL-2 cyclase domain in our *in vivo* assays. Unexpectedly, the two truncated AL-2 proteins exhibited very different properties. The #896 protein, predicted to contain a severely truncated polypeptide, exhibited a cyclase activity similar to that of the wild type enzyme. However, no cyclase activity was detected upon expression of the #897 protein under the same conditions. Both mutants have an intact cyclase domain, but the truncated PS segment of #897 interferes with the cyclase function, an effect not exhibited by the #896 protein.

Taken together, the available data show that different mutations at either of the two AL-2 domains may interfere with the activity of the other, presumably affecting protein structure or stability, providing support to the function of this bifunctional enzyme as a single polypeptide. In favor of this hypothesis, the predicted protease cleavage site found at the boundary of the cyclase and PS domains in the *P. blakesleeanus* CarRA protein is not conserved in the orthologous enzymes from other fungi, including AL-2 [21].

### Gene expression in *al-2* mutants

**Figure 8:**
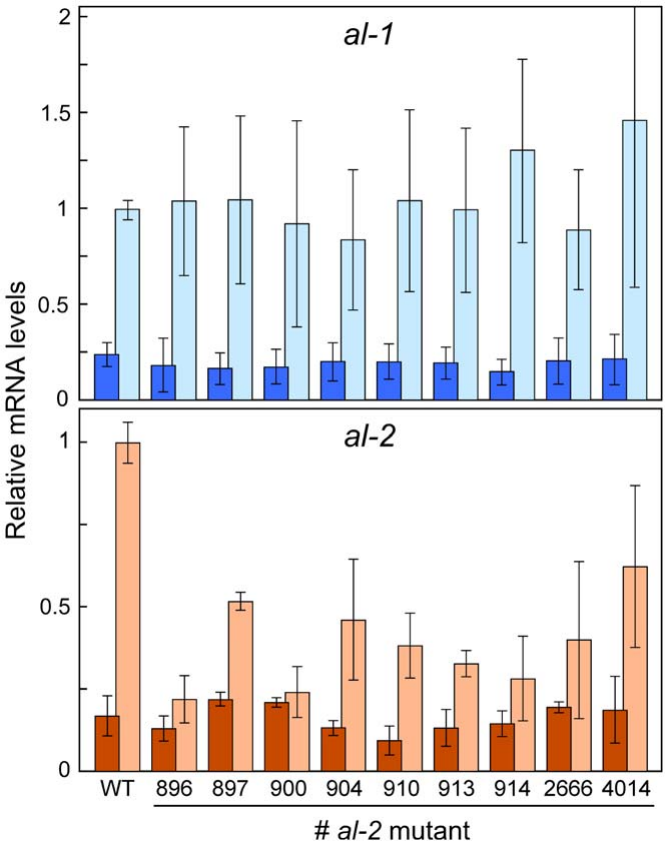
Effect of light on transcript levels of genes *al-1* and *al-2* in the wild type and *al-2* mutants. Real-time RT-PCR analyses of RNA samples isolated from the wild-type strain and the *al-2* mutants under investigation, grown in the dark (dark bars) or illuminated for 30 min (light bars). Data represent average and standard deviations of four determinations from two independent experiments. Relative mRNA levels for each gene are referred to the value from illuminated wild type samples in each set of experiments.

Neurosporaxanthin biosynthesis is induced by light in *N. crassa* mycelia by the *White Collar* photoreceptor system, which activates the transcription of the enzymatic genes [34]. Thus, lack of carotenoids in *N. crassa* mutants may also be explained by a regulatory defect in the photoinduction mechanism. We carried out experiments to determine the mRNA levels of genes *al-1* and *al-2* in the wild type strain and *al-2* mutants grown in darkness or after 30 min illumination, sufficient for a full photoresponse under our culture conditions [13]. As expected, *al-1* and *al-2* mRNA augmented in the wild type strain upon illumination. However, photoinduction of *al-2* mRNA was reduced 2 to 4-fold in the *al-2* mutants compared to the control strain while that of *al-1* was essentially unaffected (Fig. 8). This effect could be added to the imputed enzymatic defects to explain the reduced carotenoid content in these strains.

The attenuated *al-2* photoinduction in the *al-2* mutants is an unexpected result, since the presumed alterations of the AL-2 enzyme should be independent of the WC-mediated regulation of the gene. Furthermore, this effect was exhibited by *al-2* but not by *al-1*, although both genes are controlled by the same photoinduction system [35]. As a tentative explanation, *al-2* mRNA stability could be modulated by an unknown signal associated to the AL-2 enzymatic activity. Interestingly, a similar effect was formerly reported for the *al-2* orthologous gene of *P. blakesleeanus*, *carRA*: mutants of this gene also exhibited a significant reduction in the photoinduction of their own *carRA* mRNA [36]. Either in *N. crassa* or in *P. blakesleeanus*, the explanation for this regulatory effect will require further investigation.

## Materials and Methods

### Strains and growth conditions

The *Neurospora* crassa wild-type Oak Ridge 74-OR23-1A strain, the FGSC mutants listed in Table 1, and the knock-out mutants FGSC 17609 (Δ*al-1*) and FGSC 17611 (Δ*al-2*) were obtained from the Fungal Genetics Stock Center [26]. JA26 was formerly identified as a reddish *al-2* mutant with a defect in the cyclase domain [22]. Null mutations in FGSC 17609 and FGSC 17611 were generated as described [37]. The remaining mutants were obtained by exposure to UV radiation, except FGSC 2666, that was obtained by exposure to X-rays (information provided by FGSC through its mutant strain list).

For carotenoid analyses, the strains were grown in Petri dishes with 25 ml of Vogel's liquid medium [38] supplemented with 0.2% Tween 80 to suppress aerial development and conidia formation. The plates were inoculated with 10^5^ conidia and incubated for 2 days in the dark at 30°C, followed by 1 day in the light, either at 30°C or at 8°C. Illumination conditions were 4 W m^−2^ white light obtained with two fluorescent tubes. In the case of 8°C incubation, the plates were precooled in a 4°C chamber for 1 h before light exposure. For RNA extractions and subsequent cDNA generation, the strains were incubated for 1 h under light to induce *al-2* expression.

For HPLC analyses of carotenoid content in reddish *al-2* mutants, incubation and light exposure were the same, except that the cultures were grown in 250 ml Erlenmeyer flasks with 100 ml Vogel's medium. For genomic DNA extraction, the strains were grown for 2 days at 30°C in 100 ml Erlenmeyer flasks with 40 ml Vogel's medium. In both cases, incubations were done in a rotary shaker at 200 rpm.

For expression analyses, the strains were grown for 2 days at 30°C in the dark in Petri dishes with 25 ml of liquid Vogel's medium, inoculated with 10^5^ conidia, and supplemented with 0.2% Tween 80. Mycelia were collected before and after 30 min exposure to 10 W m^−2^ white light. Samples were dried on filter paper, frozen in liquid nitrogen, and stored at −80°C until use.

### Sequencing of *al-2* alleles

Genomic DNA was extracted from mycelial samples washed by filtration, frozen in liquid nitrogen, and ground into a fine powder in a cold mortar. DNA extractions were done with Genelute Plant Genomic DNA Miniprep Kit (Sigma, St Louis, MO, USA). Sequences from the wild type and nine *al-2* mutant alleles were obtained from three overlapping PCR products generated with primers 5′-CCCAAGATGTACGACTATGC-3′ and 5′-TCGAGCTTCGTCCCCGATC-3′, covering 6 bp upstream of the start codon and the first 700 bp of the *al-2* coding sequence, 5′-GACGGTGCTCCCGATGTTC-3′ and 5′-GAGTCCATCTCGAAACCCTTG-3′, covering a 750 bp internal coding segment, and 5′-AGACAGCGTTCCCTCCCTG-3′ and 5′- CACACCACATCGAACTAGCC-3′, giving a 746 bp segment extending along the last 695 bp of the coding sequence and 51 bp downstream of the stop codon. Sequences of each allele were determined from two clones obtained from independent PCR reactions, and compared with that of the wild type *Neurospora* strain (accession number L27652). DNA sequencing of PCR products was achieved by Sistemas Genómicos (Valencia, Spain). PCR reactions were performed with 50 ng genomic DNA samples of the fungal strain, 0.2 mM dNTPs, 1 µM of each primer, and 0.25 µl of the Expand PCR System (Boehringer, Mannheim, Germany). Reaction mixtures were heated at 94°C for 2 min followed by 35 cycles of denaturation (94°C, 20 s), annealing (52°C, 20 s) and polymerization (72°C, 1 min) and by a final polymerization at 72°C for 5 min in a thermocycler (Techne ftgene2d). Amplified DNA fragments were purified from agarose gels with the GFX™ PCR DNA and Gel Band Purification Kit (Amersham Biosciences, NJ, USA).

### *In vivo* assays of cyclase activity

To check cyclase activity of the selected AL-2 enzymes, corresponding cDNAs were obtained by PCR from cDNA samples synthesized from total RNA of the mutants with the SuperScript III Reverse Transcriptase kit (Invitrogen, Carlsbad, CA, USA). PCR reactions were performed with Phu Polymerase (Phusion High Fidelity DNA Polymerase, New England Biolabs, Ipswich, MA, USA), using the primers 5′-ATGTACGACTATGCTTTTGT-3′ and 5′-CACACCACATCGAACTAGCC-3′. The reactions consisted of 30 s of initial denaturation at 98°C, 32 cycles of denaturation (98°C, 15 s), annealing (58°C, 30 s) and polymerization (72°C, 90 s) and by a final polymerization at 72°C for 10 min. RNA samples were obtained with the RNeasy Plant Mini Kit (Qiagen, Hilden, Germany). The resulting PCR products were cloned in the vector pBLUNT (Zero Blunt PCR cloning Kit, Invitrogen). Lack of introns and sequence integrity, including the expected mutations, were checked by sequencing from M13 F and R primers.

The cloned cDNAs, obtained from wild type strain and mutants #896 and #897, were amplified by PCR with primers 5′-AAGCGGCCGCGCGCCCAATACGC-3′ and 5′-CGTTTCTAGAGGCACACCACATCG-3′, which contain *Not*I and *Xba*I restriction sites for generation of the *al-2* coding sequence under control of the *Escherichia coli lac* promoter. The PCR products were purified and subcloned in the plasmid pFarbeR [25], that contains three genes from *Erwinia herbicola* coding for enzymes needed for lycopene synthesis in *E. coli*: CrtE (GGPP synthesis from FPP and IPP), CrtB (phytoene synthesis from GGPP) and CrtI (lycopene synthesis from phytoene). The resulting plasmids were checked by restriction analyses and introduced by transformation in the *E. coli* TOP 10 strain (Invitrogen).

The *E. coli* transformants harboring the plasmid allowing lycopene production and expressing different *al-2* alleles were grown at 24°C in LB medium supplemented with 50 mg l^−1^ kanamycin to reach an OD_600_ of 0.6. Then, 100 µM isopropyl-β-D-thiogalactopyranoside (IPTG) was added to induce *al-2* gene expression and the cultures were incubated at 20°C overnight. Finally, the cells were collected by centrifugation and used for carotenoid extractions.

### Carotenoid analyses

The mycelial samples were dried on filter paper and lyophilized before extraction. Carotenoids were extracted by breaking the dry mycelial samples in a FastPrep® 24 device (MP Biomedicals, Irvine, CA), and analyzed as described by Arrach et al. [22]. Total carotenoid contents were estimated from their maximal absorption peaks using the extinction coefficient for neurosporaxanthin, E (1 mg l^−1^, 1 cm) = 171.5. For low carotenoid amounts (<20 µg g^−1^ dry wt), the samples were concentrated and measured in a 100 µl micro cell.

HPLC analyses of the carotenoid contents of the wild type, JA26 and #2666 were done with a Hewlett Packard 1100 series system (Waldbronn, Germany), equipped with a photodiode array detector and a C30-reversed phase column (Waters, hypersil ODS). The separations were performed using MeOH/tert-butylmethylether, 1∶1, v/v (solvent A) and MeOH/tertbutylmethylether/water, 5∶1∶1, v/v/v (solvent B). The column was developed at a flow rate of 1 ml min^−1^ with a linear gradient from 100% solvent B to 57% solvent A/43% solvent B within 45 min, followed by a linear gradient to 100% solvent A with a flow rate of 2 ml within 25 min. The flow was then increased to 2 ml min^−1^ of 100% solvent B maintaining these conditions for 11 min.

For extraction of carotenoids in *E. coli*, the cultures were centrifuged and the pellets were resuspended in 5 ml acetone and subjected to sonication. After centrifugation, epiphases were discarded and organic phases were isolated, vacuum-dried and resuspended in 80 µl CHCl_3_ for HPLC analyses. HPLC separations were achieved with a Waters system Alliance 2695 (Eschborn, Germany) equipped with a photodiode array detector (model 996) and a YMC-Pack C30-reversed phase column (250×4.6 mm i.d., 5 µm; YMC Europe, Schermbeck, Germany), using the solvents A (MeOH∶TBME∶H_2_O 5∶1∶1 v/v/v) and B (MeOH∶TBME 7∶13 v/v). The column was developed first at a flow-rate of 1 ml min^−1^ for 40 min with solvent A, then 10 min with solvents A∶B 1∶1, followed by 20 min at a flow-rate of 2 ml min^−1^ with solvent B.

### Expression analyses

Mycelia were lysed in a Fast-Prep®-24 homogenizer (MP™Biomedicals LLC Europe, France) using zirconia microbeads and 2 pulses of 30 s at 6 m s^−1^. Total RNA was isolated using the RNeasy Plant Mini kit (Qiagen, Valencia, CA, USA) and treated with rDNAse I according to the manufacturer (USB, Affymetrix, Cleveland, OH, USA). Total RNA concentration was estimated using a Nanodrop ND-1000 spectrophotometer (NanoDrop Technologies, Wilmington, DE, USA).

RT-PCR mixtures are detailed in Limón et al. [39]. RT-PCRs were performed in an ABI 7500 (Applied Biosystems, Foster City, CA, USA) using a PCR program described by Estrada et al. [13]. Genes and primer sets to analyze gene expression by RT-PCR were: *al-1* (5′-TCCAATGTTTCCCCAACTACAAC-3′ and 5′-CGGTGGTGGGCGAGAA-3), *al-2* (5′-CGCTATCGCCTACCCCATT-3′ and 5′-CGACGAGGAAGCCTGTTTG-3′), and NCU04054 (β-tubulin gene, used as endogenous control for constitutive expression, 5′-CGTCCATCAGCTCGTTGAGA-3′ and 5′-CGCCTCGTTGTCAATGCA-3′).

Primer design and relative gene expression were done as described by Rodríguez-Ortiz et al. [40]. Each RT-PCR analysis was performed four times (duplicated samples from two independent experiments). Error bars indicate standard deviation.

### Sequence analyses

Sequence alignments were achieved with the ClustalX 1.83 program [41]. *Neurospora* DNA sequences were obtained through the server www.broad.mit.edu/annotation/fungi/ of the Broad Institute (Cambridge, MA. USA). Data on residues involved in PS catalytic domains are mentioned in gene bank accession number for *al-2*, AAA19428, according to data for Trans-Isoprenyl Diphosphate Synthases, head-to-head, from the NCBI conserved domain database [42]; NCBI server: www.ncbi.nlm.nih.gov/Structure/index.shtml.

## Supporting Information

Supporting Information S1 ***al-2*** **sequences in mutant strains of** ***N. crassa*** **.**
(PDF)
