# Supplementary material for: Analysis of al-2 Mutations in Neurospora
Source: PLoS One. 2011 Jul 19;6(7):e21948. doi: 10.1371/journal.pone.0021948 (PMC3139582; doi:10.1371/journal.pone.0021948)
Supplement: Supporting Information S1 — al-2 sequences in mutant strains of N. crassa . (PDF) [file pone.0021948.s001.pdf]

Analysis of *al-2* Mutations in *Neurospora*

Violeta Díaz-Sánchez, Alejandro F. Estrada, Danika Trautmann, M. Carmen Limón, Salim Al-Babili and Javier Avalos

Supporting information 1

al-2 sequences in mutant strains of *N. crassa*

```
Start and stop codons in bold letters (increased font size)
Introns in red lower case
Mutations underlined and shaded in color
```

Wild-type *al-2* allele (from Accession No. L27652)

ATGTACGACTATGCTTTTGTgtgagtaccagatctgacagccaccaccctttagtcctgggaaacatcattgctgatga
gatacatgatagTCACTTAAAATTCACGGTACCCGCGGCGGTACTTCTCACCGCTATCGCCTACCCCATTCTCAACAGGA
TACATCTCATCCAAACAGGCTTCCTCGTCGTCGTCGCCTTTACCGCCGCTCTGCCATGGGATGCCTACTTGATTAAGCAC
AAAGTATGGTCTTACCCACCAGAAGCCATTGTTGGGCCGCGTTTGCTTGGAATTCCCTTTGAAGAGCTGTTCTTCTTTGT
GATACAGACTTACATCACGGCGCTCGTATACATCCTCTTCAACAAGCCGGTGCTGCACGCGTTGCACCTCAACAATCAAC
AAAACCCGCCAGCATGGATGAGGGTTGTCAAGGTTACCGGCCAGGTAGTCCTCGTAGCCTTGTCGGTATGGGGATGGAAT
GCCGCTCAGGTTCATCAGGAAACAAGCTATCTCGGCTTGATCCTTGTTTGGGCTTGTCCGTTCTTACTGGCTATCTGGAC
CCTCGCTGGGCGCTTCATTCTCAGCCTACCCTGGTACGCGACGGTGCTCCCGATGTTCCTACCCACCTTCTATCTTTGGG
CGGTAGACGAGTTTGCCTTGCACAGGGGTACTTGGTCCATCGGATCGGGGACGAAGCTCGATTTTTGTCTGTTTGGCAAG
TTGGACATTGAAGAAGCCACGTTCTTCCTGGTGACCAACATGCTCATCGTTGGCGGTATGGCCGCGTTCGATCAATATCT
GGCCGTCATTTACGCTTTCCCAACTCTGTTCCCCAAGGTCAACCGGTATCCGACAACTCATATGCTTCTTCAAAGCCGTC
TTATCAACACTTCCAGGTACGATCTTGAGCGCATTGAGGGCCTGAGAGAAGCGGTCGAGAGACTGCGCCTGAAGAGCAGG
AGTTTTTACCTGGCCAATTCGCTCTTTTCTGGTCGACTCCGCATTGACCTGATCCTGCTgtaagtacccattcagcctaa
gcaaagttccggacttgaactaataaatcatctcagGTACTCCTTCTGTCGCCTGGCTGATGATCTAGTCGACGACGCCA
AATCTCGCCGTGAGGTCTTGTCCTGGACCGCGAAGCTGAACCACTTCCTTGATCTGCACTACAAGGACGCGGACGCCACC
GAGGACCCCAAGAAAAAGGCGGAGCGAATCGACGCCTACATCAAGACAGCGTTCCCTCCCTGTGCCTACCAAGCCCTCCA
CCTCCTGCCCACTCACATTCTTCCTCCCAAGCCTCTTTACGATCTCATCAAGGGTTTCGAGATGGACTCTCAATTCACCT
TCCACGGTACTTCCGACTCTACGGATCTCCAATACCCCATCGCCGACGACAAGGACCTTGAGAACTACGCTATCTATGTC
GCCGGTACCGTCGGCGAGCTCTGCATCGCCCTCATCATCTACCACTGCCTGCCAGACATGTCGGACACTCAGAAGCGCGA
GCTCGAGACCGCCGCGTGCCGGATGGGCATCGCGCTGCAGTACGTCAACATCGCTCGTGACATCGTCGTCGACGCACGTA
TCGGGCGCGTTTACTTGCCTACCACCTGGCTCAAGAAGGAAGGGTTGACGCACAAGATGGTCTTGGAGAACCCCGAGGGT
CCCGAGGTCATTGAGCGGATGAGAAGACGGCTTTTGGAAAATGCGTTTGAGCTGTATGGGGGCGCGAGGCCTGAGATGCA
ACGGATACCGAGCGAGGCTAGGGGCCCGATGATTGGTGCCGTTGAAAATTACATGGCGATTGGAAGGGTGTTGAGGGAGA
GGAAGGAGGGGACGGTGTTTGTGAGGATGGAGGGGAGGGCTACGGTCCCGAAGCGAAGGAGGTTGAGCACGCTGTTGAGG
GCGTTGTATGAGCAG**TAG**

Mutant alleles

FGSC896 (Accession No. FR745430)
Replacement of gt by AA; intron splicing site is changed (see text)

ATGTACGACTATGCTTTTGTgtgagtaccagatctgacagccaccaccctttagtcctgggaaacatcattgctgatga
gatacatgatagTCACTTAAAATTCACGGTACCCGCGGCGGTACTTCTCACCGCTATCGCCTACCCCATTCTCAACAGGA
TACATCTCATCCAAACAGGCTTCCTCGTCGTCGTCGCCTTTACCGCCGCTCTGCCATGGGATGCCTACTTGATTAAGCAC
AAAGTATGGTCTTACCCACCAGAAGCCATTGTTGGGCCGCGTTTGCTTGGAATTCCCTTTGAAGAGCTGTTCTTCTTTGT
GATACAGACTTACATCACGGCGCTCGTATACATCCTCTTCAACAAGCCGGTGCTGCACGCGTTGCACCTCAACAATCAAC
AAAACCCGCCAGCATGGATGAGGGTTGTCAAGGTTACCGGCCAGGTAGTCCTCGTAGCCTTGTCGGTATGGGGATGGAAT
GCCGCTCAGGTTCATCAGGAAACAAGCTATCTCGGCTTGATCCTTGTTTGGGCTTGTCCGTTCTTACTGGCTATCTGGAC
CCTCGCTGGGCGCTTCATTCTCAGCCTACCCTGGTACGCGACGGTGCTCCCGATGTTCCTACCCACCTTCTATCTTTGGG
CGGTAGACGAGTTTGCCTTGCACAGGGGTACTTGGTCCATCGGATCGGGGACGAAGCTCGATTTTTGTCTGTTTGGCAAG
TTGGACATTGAAGAAGCCACGTTCTTCCTGGTGACCAACATGCTCATCGTTGGCGGTATGGCCGCGTTCGATCAATATCT
GGCCGTCATTTACGCTTTCCCAACTCTGTTCCCCAAGGTCAACCGGTATCCGACAACTCATATGCTTCTTCAAAGCCGTC
TTATCAACACTTCCAGGTACGATCTTGAGCGCATTGAGGGCCTGAGAGAAGCGGTCGAGAGACTGCGCCTGAAGAGCAGG
AGTTTTTACCTGGCCAATTCGCTCTTTTCTGGTCGACTCCGCATTGACCTGATCCTGCTAAAAgtacccattcagcctaa
gcaaagttccggacttgaactaataaatcatctcagGTACTCCTTCTGTCGCCTGGC**TGA**TGATCTAGTCGACGACGCC
AAATCTCGCCGTGAGGTCTTGTCCTGGACCGCGAAGCTGAACCACTTCCTTGATCTGCACTACAAGGACGCGGACGCCAC
CGAGGACCCCAAGAAAAAGGCGGAGCGAATCGACGCCTACATCAAGACAGCGTTCCCTCCCTGTGCCTACCAAGCCCTCC
ACCTCCTGCCCACTCACATTCTTCCTCCCAAGCCTCTTTACGATCTCATCAAGGGTTTCGAGATGGACTCTCAATTCACC
TTCCACGGTACTTCCGACTCTACGGATCTCCAATACCCCATCGCCGACGACAAGGACCTTGAGAACTACGCTATCTATGT
CGCCGGTACCGTCGGCGAGCTCTGCATCGCCCTCATCATCTACCACTGCCTGCCAGACATGTCGGACACTCAGAAGCGCG
AGCTCGAGACCGCCGCGTGCCGGATGGGCATCGCGCTGCAGTACGTCAACATCGCTCGTGACATCGTCGTCGACGCACGT
ATCGGGCGCGTTTACTTGCCTACCACCTGGCTCAAGAAGGAAGGGTTGACGCACAAGATGGTCTTGGAGAACCCCGAGGG
TCCCGAGGTCATTGAGCGGATGAGAAGACGGCTTTTGGAAAATGCGTTTGAGCTGTATGGGGGCGCGAGGCCTGAGATGC
AACGGATACCGAGCGAGGCTAGGGGCCCGATGATTGGTGCCGTTGAAAATTACATGGCGATTGGAAGGGTGTTGAGGGAG
AGGAAGGAGGGGACGGTGTTTGTGAGGATGGAGGGGAGGGCTACGGTCCCGAAGCGAAGGAGGTTGAGCACGCTGTTGAG
GGCGTTGTATGAGCAGTAG

FGSC897 (Accession No. FR745431)
Insertion of CAAGA

ATGTACGACTATGCTTTTGTgtgagtaccagatctgacagccaccaccctttagtcctgggaaacatcattgctgatga
gatacatgatagTCACTTAAAATTCACGGTACCCGCGGCGGTACTTCTCACCGCTATCGCCTACCCCATTCTCAACAGGA
TACATCTCATCCAAACAGGCTTCCTCGTCGTCGTCGCCTTTACCGCCGCTCTGCCATGGGATGCCTACTTGATTAAGCAC
AAAGTATGGTCTTACCCACCAGAAGCCATTGTTGGGCCGCGTTTGCTTGGAATTCCCTTTGAAGAGCTGTTCTTCTTTGT
GATACAGACTTACATCACGGCGCTCGTATACATCCTCTTCAACAAGCCGGTGCTGCACGCGTTGCACCTCAACAATCAAC
AAAACCCGCCAGCATGGATGAGGGTTGTCAAGGTTACCGGCCAGGTAGTCCTCGTAGCCTTGTCGGTATGGGGATGGAAT
GCCGCTCAGGTTCATCAGGAAACAAGCTATCTCGGCTTGATCCTTGTTTGGGCTTGTCCGTTCTTACTGGCTATCTGGAC
CCTCGCTGGGCGCTTCATTCTCAGCCTACCCTGGTACGCGACGGTGCTCCCGATGTTCCTACCCACCTTCTATCTTTGGG
CGGTAGACGAGTTTGCCTTGCACAGGGGTACTTGGTCCATCGGATCGGGGACGAAGCTCGATTTTTGTCTGTTTGGCAAG
TTGGACATTGAAGAAGCCACGTTCTTCCTGGTGACCAACATGCTCATCGTTGGCGGTATGGCCGCGTTCGATCAATATCT
GGCCGTCATTTACGCTTTCCCAACTCTGTTCCCCAAGGTCAACCGGTATCCGACAACTCATATGCTTCTTCAAAGCCGTC
TTATCAACACTTCCAGGTACGATCTTGAGCGCATTGAGGGCCTGAGAGAAGCGGTCGAGAGACTGCGCCTGAAGAGCAGG
AGTTTTTACCTGGCCAATTCGCTCTTTTCTGGTCGACTCCGCATTGACCTGATCCTGCTgtaagtacccattcagcctaa
gcaaagttccggacttgaactaataaatcatctcagGTACTCCTTCTGTCGCCTGGCTGATGATCTAGTCGACGACGCCA
AATCTCGCCGTGAGGTCTTGTCCTGGACCGCGAAGCTGAACCACTTCCTTGATCTGCACTACAAGGACGCGGACGCCACC
GAGGACCCCAAGAAAAAGGCGGAGCGAATCGACGCCTACATCAAGACAGCGTTCCCTCCCTGTGCCTACCAAGCCCTCCA
CCTCCTGCCCACTCACATTCTTCCTCCCAAGCCTCTTTACGATCTCATCAAGGGTTTCGAGATGGACTCTCAATTCACCT
TCCACGGTACTTCCGACTCTACGGATCTCCAATACCCCATCGCCGACGACAAGGACCTTGAGAACTACGCTATCTATGTC
GCCGGTACCGTCGGCGAGCTCTGCATCGCCCTCATCATCTACCACTGCCTGCCAGACATGTCGGACACTCAGAAGCGCGA
GCTCGAGACCGCCGCGTGCCGGATGGGCATCGCGCTGCAGTACGTCAACATCGCTCGTGACATCGTCGTCGACGCACGTA
TCGGGCGCGTTTACTTGCCTACCACCTGGCTCAAGACAAGAAGGAAGGGT**TGA**CGCACAAGATGGTCTTGGAGAACCCC
GAGGGTCCCGAGGTCATTGAGCGGATGAGAAGACGGCTTTTGGAAAATGCGTTTGAGCTGTATGGGGGCGCGAGGCCTGA
GATGCAACGGATACCGAGCGAGGCTAGGGGCCCGATGATTGGTGCCGTTGAAAATTACATGGCGATTGGAAGGGTGTTGA
GGGAGAGGAAGGAGGGGACGGTGTTTGTGAGGATGGAGGGGAGGGCTACGGTCCCGAAGCGAAGGAGGTTGAGCACGCTG
TTGAGGGCGTTGTATGAGCAGTAG

FGSC900 (Accession No. FR745435)
Identical sequence in FGSC910
Replacement of A by C

ATGTACGACTATGCTTTTGTgtgagtaccagatctgacagccaccaccctttagtcctgggaaacatcattgctgatga
gatacatgatagTCACTTAAAATTCACGGTACCCGCGGCGGTACTTCTCACCGCTATCGCCTACCCCATTCTCAACAGGA
TACATCTCATCCAAACAGGCTTCCTCGTCGTCGTCGCCTTTACCGCCGCTCTGCCATGGGATGCCTACTTGATTAAGCAC
AAAGTATGGTCTTACCCACCAGAAGCCATTGTTGGGCCGCGTTTGCTTGGAATTCCCTTTGAAGAGCTGTTCTTCTTTGT
GATACAGACTTACATCACGGCGCTCGTATACATCCTCTTCAACAAGCCGGTGCTGCACGCGTTGCACCTCAACAATCAAC
AAAACCCGCCAGCATGGATGAGGGTTGTCAAGGTTACCGGCCAGGTAGTCCTCGTAGCCTTGTCGGTATGGGGATGGAAT
GCCGCTCAGGTTCATCAGGAAACAAGCTATCTCGGCTTGATCCTTGTTTGGGCTTGTCCGTTCTTACTGGCTATCTGGAC
CCTCGCTGGGCGCTTCATTCTCAGCCTACCCTGGTACGCGACGGTGCTCCCGATGTTCCTACCCACCTTCTATCTTTGGG
CGGTAGACGAGTTTGCCTTGCACAGGGGTACTTGGTCCATCGGATCGGGGACGAAGCTCGATTTTTGTCTGTTTGGCAAG
TTGGACATTGAAGAAGCCACGTTCTTCCTGGTGACCAACATGCTCATCGTTGGCGGTATGGCCGCGTTCGATCAATATCT
GGCCGTCATTTACGCTTTCCCAACTCTGTTCCCCAAGGTCAACCGGTATCCGACAACTCATATGCTTCTTCAAAGCCGTC
TTATCAACACTTCCAGGTACGATCTTGAGCGCATTGAGGGCCTGAGAGAAGCGGTCGAGAGACTGCGCCTGAAGAGCAGG
AGTTTTTACCTGGCCAATTCGCTCTTTTCTGGTCGACTCCGCATTGACCTGATCCTGCTgtaagtacccattcagcctaa
gcaaagttccggacttgaactaataaatcatctcagGTCCTCCTTCTGTCGCCTGGCTGATGATCTAGTCGACGACGCCA
AATCTCGCCGTGAGGTCTTGTCCTGGACCGCGAAGCTGAACCACTTCCTTGATCTGCACTACAAGGACGCGGACGCCACC
GAGGACCCCAAGAAAAAGGCGGAGCGAATCGACGCCTACATCAAGACAGCGTTCCCTCCCTGTGCCTACCAAGCCCTCCA
CCTCCTGCCCACTCACATTCTTCCTCCCAAGCCTCTTTACGATCTCATCAAGGGTTTCGAGATGGACTCTCAATTCACCT
TCCACGGTACTTCCGACTCTACGGATCTCCAATACCCCATCGCCGACGACAAGGACCTTGAGAACTACGCTATCTATGTC
GCCGGTACCGTCGGCGAGCTCTGCATCGCCCTCATCATCTACCACTGCCTGCCAGACATGTCGGACACTCAGAAGCGCGA
GCTCGAGACCGCCGCGTGCCGGATGGGCATCGCGCTGCAGTACGTCAACATCGCTCGTGACATCGTCGTCGACGCACGTA
TCGGGCGCGTTTACTTGCCTACCACCTGGCTCAAGAAGGAAGGGTTGACGCACAAGATGGTCTTGGAGAACCCCGAGGGT
CCCGAGGTCATTGAGCGGATGAGAAGACGGCTTTTGGAAAATGCGTTTGAGCTGTATGGGGGCGCGAGGCCTGAGATGCA
ACGGATACCGAGCGAGGCTAGGGGCCCGATGATTGGTGCCGTTGAAAATTACATGGCGATTGGAAGGGTGTTGAGGGAGA
GGAAGGAGGGGACGGTGTTTGTGAGGATGGAGGGGAGGGCTACGGTCCCGAAGCGAAGGAGGTTGAGCACGCTGTTGAGG
GCGTTGTATGAGCAG**TAG**

FGSC904 (Accession No. FR745432)
Replacement of GAA by AG

ATGTACGACTATGCTTTTGTgtgagtaccagatctgacagccaccaccctttagtcctgggaaacatcattgctgatga
gatacatgatagTCACTTAAAATTCACGGTACCCGCGGCGGTACTTCTCACCGCTATCGCCTACCCCATTCTCAACAGGA
TACATCTCATCCAAACAGGCTTCCTCGTCGTCGTCGCCTTTACCGCCGCTCTGCCATGGGATGCCTACTTGATTAAGCAC
AAAGTATGGTCTTACCCACCAGAAGCCATTGTTGGGCCGCGTTTGCTTGGAATTCCCTTTGAAGAGCTGTTCTTCTTTGT
GATACAGACTTACATCACGGCGCTCGTATACATCCTCTTCAACAAGCCGGTGCTGCACGCGTTGCACCTCAACAATCAAC
AAAACCCGCCAGCATGGATGAGGGTTGTCAAGGTTACCGGCCAGGTAGTCCTCGTAGCCTTGTCGGTATGGGGATGGAAT
GCCGCTCAGGTTCATCAGGAAACAAGCTATCTCGGCTTGATCCTTGTTTGGGCTTGTCCGTTCTTACTGGCTATCTGGAC
CCTCGCTGGGCGCTTCATTCTCAGCCTACCCTGGTACGCGACGGTGCTCCCGATGTTCCTACCCACCTTCTATCTTTGGG
CGGTAGACGAGTTTGCCTTGCACAGGGGTACTTGGTCCATCGGATCGGGGACGAAGCTCGATTTTTGTCTGTTTGGCAAG
TTGGACATTGAAGAAGCCACGTTCTTCCTGGTGACCAACATGCTCATCGTTGGCGGTATGGCCGCGTTCGATCAATATCT
GGCCGTCATTTACGCTTTCCCAACTCTGTTCCCCAAGGTCAACCGGTATCCGACAACTCATATGCTTCTTCAAAGCCGTC
TTATCAACACTTCCAGGTACGATCTTGAGCGCATTGAGGGCCTGAGAGAAGCGGTCGAGAGACTGCGCCTGAAGAGCAGG
AGTTTTTACCTGGCCAATTCGCTCTTTTCTGGTCGACTCCGCATTGACCTGATCCTGCTgtaagtacccattcagcctaa
gcaaagttccggacttgaactaataaatcatctcagGTACTCCTTCTGTCGCCTGGCTGATGATCTAGTCGACGACGCCA
AATCTCGCCGTGAGGTCTTGTCCTGGACCGCGAAGCTGAACCACTTCCTTGATCTGCACTACAAGGACGCGGACGCCACC
GAGGACCCCAAAGAAAGGCGGAGCGAATCGACGCCTACATCAAGACAGCGTTCCCTCCCTGTGCCTACCAAGCCCTCCAC
CTCCTGCCCACTCACATTCTTCCTCCCAAGCCTCTTTACGATCTCATCAAGGGTTTCGAGATGGACTCTCAATTCACCTT
CCACGGTACTTCCGACTCTACGGATCTCCAATACCCCATCGCCGACGACAAGGACCTTGAGAACTACGCTATCTATGTCG
CCGGTACCGTCGGCGAGCTCTGCATCGCCCTCATCATCTACCACTGCCTGCCAGACATGTCGGACACTCAGAAGCGCGAG
CTCGAGACCGCCGCGTGCCGGATGGGCATCGCGCTGCAGTACGTCAACATCGCTCGTGACATCGTCGTCGACGCACGTAT
CGGGCGCGTTTACTTGCCTACCACCTGGCTCAAGAAGGAAGGGT**TGA**CGCACAAGATGGTCTTGGAGAACCCCGAGGGT
CCCGAGGTCATTGAGCGGATGAGAAGACGGCTTTTGGAAAATGCGTTTGAGCTGTATGGGGGCGCGAGGCCTGAGATGCA
ACGGATACCGAGCGAGGCTAGGGGCCCGATGATTGGTGCCGTTGAAAATTACATGGCGATTGGAAGGGTGTTGAGGGAGA
GGAAGGAGGGGACGGTGTTTGTGAGGATGGAGGGGAGGGCTACGGTCCCGAAGCGAAGGAGGTTGAGCACGCTGTTGAGG
GCGTTGTATGAGCAGTAG

FGSC913 (Accession No. FR745433)
Deletion of (T)

ATGTACGACTATGCTTTTGTgtgagtaccagatctgacagccaccaccctttagtcctgggaaacatcattgctgatga
gatacatgatagTCACTTAAAATTCACGGTACCCGCGGCGGTACTTCTCACCGCTATCGCCTACCCCATTCTCAACAGGA
TACATCTCATCCAAACAGGCTTCCTCGTCGTCGTCGCCTTTACCGCCGCTCTGCCATGGGATGCCTACTTGATTAAGCAC
AAAGTATGGTCTTACCCACCAGAAGCCATTGTTGGGCCGCGTTTGCTTGGAATTCCCTTTGAAGAGCTGTTCTTCTTTGT
GATACAGACTTACATCACGGCGCTCGTATACATCCTCTTCAACAAGCCGGTGCTGCACGCGTTGCACCTCAACAATCAAC
AAAACCCGCCAGCATGGATGAGGGTTGTCAAGGTTACCGGCCAGGTAGTCCTCGTAGCCTTGTCGGTATGGGGATGGAAT
GCCGCTCAGGTTCATCAGGAAACAAGCTATCTCGGCTTGATCCTTGTTTGGGCTTGTCCGTTCTTACTGGCTATCTGGAC
CCTCGCTGGGCGCTTCATTCTCAGCCTACCCTGGTACGCGACGGTGCTCCCGATGTTCCTACCCACCTTCTATCTTTGGG
CGGTAGACGAGTTTGCCTTGCACAGGGGTACTTGGTCCATCGGATCGGGGACGAAGCTCGATTTTTGTCTGTTTGGCAAG
TTGGACATTGAAGAAGCCACGTTCTTCCTGGTGACCAACATGCTCATCGTTGGCGGTATGGCCGCGTTCGATCAATATCT
GGCCGTCATTTACGCTTTCCCAACTCTGTTCCCCAAGGTCAACCGGTATCCGACAACTCATATGCTTCTTCAAAGCCGTC
TTATCAACACTTCCAGGTACGATCTTGAGCGCATTGAGGGCCTGAGAGAAGCGGTCGAGAGACTGCGCCTGAAGAGCAGG
AGTTTTTACCTGGCCAATTCGCTCTTTTCTGGTCGACTCCGCATTGACCTGATCCTGCTgtaagtacccattcagcctaa
gcaaagttccggacttgaactaataaatcatctcagGTACTCCTTCTGTCGCCTGGCTGATGATCTAGTCGACGACGCCA
AATCTCGCCGTGAGGTCTTGTCCTGGACCGCGAAGCTGAACCACTTCCTTGATCTGCACTACAAGGACGCGGACGCCACC
GAGGACCCCAAGAAAAAGGCGGAGCGAATCGACGCC(T)ACATCAAGACAGCGTTCCCTCCCTGTGCCTACCAAGCCCTC
CACCTCCTGCCCACTCACATTCTTCCTCCCAAGCCTCTTTACGATCTCATCAAGGGTTTCGAGATGGACTCTCAATTCAC
CTTCCACGGTACTTCCGACTCTACGGATCTCCAATACCCCATCGCCGACGACAAGGACCTTGAGAACTACGCTATCTATG
TCGCCGGTACCGTCGGCGAGCTCTGCATCGCCCTCATCATCTACCACTGCCTGCCAGACATGTCGGACACTCAGAAGCGC
GAGCTCGAGACCGCCGCGTGCCGGATGGGCATCGCGCTGCAGTACGTCAACATCGCTCGTGACATCGTCGTCGACGCACG
TATCGGGCGCGTTTACTTGCCTACCACCTGGCTCAAGAAGGAAGGGT**TGA**CGCACAAGATGGTCTTGGAGAACCCCGAG
GGTCCCGAGGTCATTGAGCGGATGAGAAGACGGCTTTTGGAAAATGCGTTTGAGCTGTATGGGGGCGCGAGGCCTGAGAT
GCAACGGATACCGAGCGAGGCTAGGGGCCCGATGATTGGTGCCGTTGAAAATTACATGGCGATTGGAAGGGTGTTGAGGG
AGAGGAAGGAGGGGACGGTGTTTGTGAGGATGGAGGGGAGGGCTACGGTCCCGAAGCGAAGGAGGTTGAGCACGCTGTTG
AGGGCGTTGTATGAGCAGTAG

FGSC914 (Accession No. FR745434)
Replacement of G by A

ATGTACGACTATGCTTTTGTgtgagtaccagatctgacagccaccaccctttagtcctgggaaacatcattgctgatga
gatacatgatagTCACTTAAAATTCACGGTACCCGCGGCGGTACTTCTCACCGCTATCGCCTACCCCATTCTCAACAGGA
TACATCTCATCCAAACAGGCTTCCTCGTCGTCGTCGCCTTTACCGCCGCTCTGCCATGGGATGCCTACTTGATTAAGCAC
AAAGTATGGTCTTACCCACCAGAAGCCATTGTTGGGCCGCGTTTGCTTGGAATTCCCTTTGAAGAGCTGTTCTTCTTTGT
GATACAGACTTACATCACGGCGCTCGTATACATCCTCTTCAACAAGCCGGTGCTGCACGCGTTGCACCTCAACAATCAAC
AAAACCCGCCAGCATGGATGAGGGTTGTCAAGGTTACCGGCCAGGTAGTCCTCGTAGCCTTGTCGGTATGGGGATGGAAT
GCCGCTCAGGTTCATCAGGAAACAAGCTATCTCGGCTTGATCCTTGTTTGGGCTTGTCCGTTCTTACTGGCTATCTGGAC
CCTCGCTGGGCGCTTCATTCTCAGCCTACCCTGGTACGCGACGGTGCTCCCGATGTTCCTACCCACCTTCTATCTTTGGG
CGGTAGACGAGTTTGCCTTGCACAGGGGTACTTGGTCCATCGGATCGGGGACGAAGCTCGATTTTTGTCTGTTTGGCAAG
TTGGACATTGAAGAAGCCACGTTCTTCCTGGTGACCAACATGCTCATCGTTGGCGGTATGGCCGCGTTCGATCAATATCT
GGCCGTCATTTACGCTTTCCCAACTCTGTTCCCCAAGGTCAACCGGTATCCGACAACTCATATGCTTCTTCAAAGCCGTC
TTATCAACACTTCCAGGTACGATCTTGAGCGCATTGAGGGCCTGAGAGAAGCGGTCGAGAGACTGCGCCTGAAGAGCAGG
AGTTTTTACCTGGCCAATTCGCTCTTTTCTGGTCGACTCCGCATTGACCTGATCCTGCTgtaagtacccattcagcctaa
gcaaagttccggacttgaactaataaatcatctcagGTACTCCTTCTGTCGCCTGGCTGATGATCTAGTCGACGACGCCA
AATCTCGCCGTGAGGTCTTGTCC**TGA**ACCGCGAAGCTGAACCACTTCCTTGATCTGCACTACAAGGACGCGGACGCCAC
CGAGGACCCCAAGAAAAAGGCGGAGCGAATCGACGCCTACATCAAGACAGCGTTCCCTCCCTGTGCCTACCAAGCCCTCC
ACCTCCTGCCCACTCACATTCTTCCTCCCAAGCCTCTTTACGATCTCATCAAGGGTTTCGAGATGGACTCTCAATTCACC
TTCCACGGTACTTCCGACTCTACGGATCTCCAATACCCCATCGCCGACGACAAGGACCTTGAGAACTACGCTATCTATGT
CGCCGGTACCGTCGGCGAGCTCTGCATCGCCCTCATCATCTACCACTGCCTGCCAGACATGTCGGACACTCAGAAGCGCG
AGCTCGAGACCGCCGCGTGCCGGATGGGCATCGCGCTGCAGTACGTCAACATCGCTCGTGACATCGTCGTCGACGCACGT
ATCGGGCGCGTTTACTTGCCTACCACCTGGCTCAAGAAGGAAGGGTTGACGCACAAGATGGTCTTGGAGAACCCCGAGGG
TCCCGAGGTCATTGAGCGGATGAGAAGACGGCTTTTGGAAAATGCGTTTGAGCTGTATGGGGGCGCGAGGCCTGAGATGC
AACGGATACCGAGCGAGGCTAGGGGCCCGATGATTGGTGCCGTTGAAAATTACATGGCGATTGGAAGGGTGTTGAGGGAG
AGGAAGGAGGGGACGGTGTTTGTGAGGATGGAGGGGAGGGCTACGGTCCCGAAGCGAAGGAGGTTGAGCACGCTGTTGAG
GGCGTTGTATGAGCAGTAG

FGSC2666 (Accession No. FR745436)
19 point mutations; silent mutations in yellow

ATGTACGACTATGCTTTTGTgtgagtaccagatctgacagccaccaccctttagtcctgggaaacatcattgctgatga
gatacatgatagTCACTTAAAATTCACGGTACCCGTGGCGGTACTTCTCACCGCTATCGCCTACCCCATTCTCAACAGGA
TACATCTCATCCAAACAGGCTTCCTCGTCGTCGTCGCCTTTACCGCCGCTCTGCCATGGGATGCCTACTTGATTAAGCAC
AAAGTATGGTCTGACCCACCAGAAGCCATTGTTGGGCCGCGTTTGCTTGGAATTCCCTTTGAAGAGCTGTTCTTCTTTGT
GATACAGACTTACATCACGGCGCTCGTATACATCCTCTTCAACAAGCCGGTGCTGCACGCGTTGCACCTCAACAATCAAC
AAAACCCGCCAGCGTGGATGAGGGTTGTCAAGGTTACCGGTCAGGTAGTCCTCGTAGCCTTGTCGGTATGGGGATGGAAT
GCCGCTCAGGTTCATCAGGAAACAAGCTATCTCGGCTTGATCCTTGTTTGGGCTTGTCCGTTCTTACTGGCTATCTGGAC
CCTCGCTGGGCGCTTCATTCTCAGCCTACCATGGTACGCGACGCTGCTCCCGATGTTCCTACCCACCTTCTATCTTTGGG
CGGTAGACGAGTTTGCCTTGCACAGGGGTACTTGGTCCATCGGATCGGGGACGAAGCTCGATTTTTGTCTGTTTGGCAAG
TTGGACATTGAAGAAGCCACGTTCTTCCTGGTGACCAACATGCTCATCGTTGGCGGTATGGCCGCGTTCGATCAATACCT
GGCCGTCATTTACGCTTTCCCAACGCTGTTCCCCAAGGTCAACCGGTATCCGACACCTCATATGCTTCTTCAAAGCCGTC
TTATCAACACTTCCAGGTACGATCTTGAGCGCATTGAGGGTCTGAGAGAAGCGGTCGAGAGACTGCGCCTGAAGAGCAGG
AGTTTTTACCTGGCCAATTCGCTCTTTTCTGGTCGACTCCGCATTGACCTGATCCTGCTgtaagtacccattcagcctaa
ccaaagttccggacttgaactaataaaccatctcagGTACTCCTTCTGTCGCCTGGCTGATGATCTAGTCGACGACGCTA
AATCTCGCCGCGAGGTCTTGTCCTGGACCGCGAAGCTGAACCACTTCCTTGATCTGCACTACAAGGACGCGGACGCCACC
GAGGACCCCAAGAAAAAGGCGGAGCGAATCGACGCCTACATCAAGACAGCGTTCCCTCCCTGTGCCTACCAAGCCCTCCA
CCTCCTGCCCACTCACATTCTTCCTCCCAAGCCTCTTTACGATCTCATCAAGGGTTTCGAGATGGACTCTCAATTCACCT
TCCACGGTACTTCTGACTCTACGGATCTCCAATACCCCATCGCCGACGACAAGGACCTTGAGAACTACGCTATCTATGTC
GCCGGCACCGTCGGCGAGCTCTGCATCGCCCTCATCATCTACCACTGCCTGCCAGACATGTCGGACACTCAGAAGCGCGA
GCTCGAGACCGCCGCGTGCCGGATGGGCATTGCGCTGCAGTACGTCAACATCGCTCGCGACATCGTCGTCGACGCACGTA
TTGGGCGCGTTTACTTGCCTACCACCTGGCTCAAGAAGGAAGGGTTGACGCACAAGATGGTCTTGGAGAACCCCGAGGGT
CCCGAGGTCATTGAGCGGATGAGAAGACGGCTTTTGGAAAATGCGTTTGAGCTGTATGGGGGCGCGAGGCCTGAGATGCA
ACGGATACCGAGCGAGGCTAGGGGCCCGATGATTGGTGCCGAGGAAAATTACATGGCGATTGGAAGGGTGTTGAGGGAGA
GGAAGGAGGGGACGGTGTTTGTGAGGATGGAGGGGAGGGCTACGGTCCCGAAGCGAAGGAGGTTGAGCACGCTGTTGAGG
GCGTTGTATGAGCAG**TAG**

FGSC4014 (Accession No. FR745437)
11 point mutations; silent mutations in yellow

ATGTACGACTATGCTTTTGTgtgagtaccagatctgacagccaccaccctttagtcctgggaaacatcattgctgatga
gatacatgatagTCACTTAAAATTCACGGTACCCGCGGCGGTACTTCTCACCGCTATCGCCTACCCCATTCTCAACAGGA
TACATCTCATCCAAACAGGCTTCCTCGTCGTCGTCGCCTTTACCGCCGCTCTGCCATGGGATGCCTACTTGATTAAGCAC
AAAGTATGGTCTTACCCACCAGAAGCCATTGTTGGGCCGCGTTTGCTTGGAATTCCCTTTGAAGAGCTGTTCTTCTTTGT
GATACAGACTTACATCACGGCGCTCGTATACATCCTCTTCAACAAGCCGGTGCTGCACGCGTTGCACCTCAACAATCAAC
AAAACCCGCCAGCATGGATGAGGGTTGTCAAGGTTACCGGCCAGGTAGTCCTCGTAGCCTTGTCGGTATGGGGATGGAAT
GCCGCTCAGGTTCATCAGGAAACAAGCTATCTCGGCTTGATCCTTGTTTGGGCTTGTCCGTTCTTACTGGCTATCTGGAC
CCTCGCTGGGCGCTTCATTCTCAGCCTACCCTGGTACGCGACGGTGCTCCCGATGTTCCTACCCACCTTCTATCTTTGGG
CGGTAGACGAGTTTGCCTTGCACAGGGGTACTTGGTCCATCGGATCGGGGACGAAGCTCGATTTTTGTCTGTTTGGCAAG
TTGGACATTGAAGAAGCCACGTTCTTCCTGGTGACCAACATGCTCATCGTTGGCGGTATGGCCGCGTTCGATCAATATCT
GGCCGTCATTTACGCTTTCCCAACTCTGTTCCCCAAGGTCAACCGGTATCCGACAGCTCATATGCTTCTTCAAAGCCGTC
TTATCAACACTTCCAGGTACGATCTTGAGCGCATTGAGGGTCTGAGAGAAGCGGTCGAGAGACTGCGCCTGAAGAGCAGG
AGTTTTTACCTGGCCAATTCGCTCTTTTCTGGTCGACTCCGCATTGACCTGATCCTGCTgtaagtacccattcagcctaa
ccaaagttccggacttgaactaataaatcatctcagGTACTCCTTCTGTCGCCTGGCTGATGATCTAGTCGACGACGCTA
AATCTCGCCGCGAGGTCTTGTCCTGGACCGCGAAGCTGAACCACTTCCTTGATCTGCACTACAAGGACGCGGACGCCACC
GAGGACCCCAAGAAAAAGGCGGAGCGAATCGACGCCTACATCAAGACAGCGTTCCCTCCCTGTGCCTACCAAGCCCTCCA
CCTCCTGCCCACTCACATTCTTCCTCCCAAGCCTCTTTACGATCTCATCAAGGGTTTCGAGATGGACTCTCAATTCACCT
TCCACGGTACTTCTGACTCTACGGATCTCCAATACCCCATCGCCGACGACAAGGACCTTGAGAACTACGCTATCTATGTC
GCCGGCACCGTCGGCGAGCTCTGCATCGCCCTCATCATCTACCACTGCCTGCCAGACATGTCGGACACTCAGAAGCGCGA
GCTCGAGACCGCCGCGTGCCGGATGGGCATTGCGCTGCAGTACGTCAACATCGCTCGCGACATCGTCGTCGACGCACGTA
TTGGGCGCGTTTACTTGCCTACCACCTGGCTCAAGAAGGAAGGGTTGACGCACAAGATGGTCTTGGAGAACCCCGAGGGT
CCCGAGGTCATTGAGCGGATGAGAAGACGGCTTTTGGAAAATGCGTTTGAGCTGTATGGGGGCGCGAGGCCTGAGATGCA
ACGGATACCGAGCGAGGCTAGGGGCCCGATGATTGGTGCCGAGGAAAATTACATGGCGATTGGAAGGGTGTTGAGGGAGA
GGAAGGAGGGGACGGTGTTTGTGAGGATGGAGGGGAGGGCTACGGTCCCGAAGCGAAGGAGGTTGAGCACGCTGTTGAGG
GCGTTGTATGAGCAG**TAG**
